# Adult‐Onset Dystonia Related to *PRKRA* (DYT16): A Predictor of Favorable Deep Brain Stimulation Outcome

**DOI:** 10.1002/mdc3.70837

**Published:** 2026-09-27

**Authors:** Ingrid Lorena da Silva Gomes, Eduardo Drews Amorim, Juan Sebastián Sánchez León, Thomas Hugentobler Schlickmann, Yuri Ferreira Felloni Borges, Artur Francisco Schumacher Schuh, Daniel Teixeira‐dos‐Santos, Sheila Trentin, Thais Lampert Monte, Bruno Samuel Fraiman de Oliveira

**Affiliations:** ^1^ Department of Neurology Hospital de Clínicas de Porto Alegre Porto Alegre Brazil; ^2^ Universidade Federal do Rio Grande do Sul; ^3^ Hospital São Lucas da PUCRS

**Keywords:** DBS, dystonia, DYT16, DYT‐*PRKRA*

Dystonia comprises a heterogeneous group of movement disorders characterized by sustained or intermittent abnormal movements and/or postures.^1^, ^2^ Accurate classification of dystonia is essential for both etiological investigation and the selection of optimal treatment strategies. Although focal and segmental forms often respond to botulinum toxin injections, generalized dystonia remains particularly challenging to manage.^3^

Advances in molecular genetics have expanded our understanding of inherited dystonias, several of which demonstrate variable but often substantial responsiveness to deep brain stimulation (DBS) when pharmacological therapy fails. The most consistent benefit has been reported in *TOR1A* (DYT1) followed by *SGCE*, *THAP1*, *KMT2B*, and *VPS16*. Other genotypes such as *PRKRA*, *ATP1A3*, and *GNAL* display heterogeneous responses influenced by phenotype, disease duration, and age at intervention.^3^

Autosomal recessive dystonia caused by biallelic *PRKRA* mutations (DYT16) was first described in Brazilian families, most commonly presenting as juvenile‐onset generalized dystonia with parkinsonian features.^4^ Although additional cases have been reported in Europe,^5^ DYT16 remains rare, accounting for approximately 4.5% of idiopathic dystonia in a Brazilian cohort.^6^ Clinical expression varies both within and between families. Levodopa (l‐dopa) responsiveness is typically absent, and treatment options are limited. Importantly, the determinants of DBS outcomes in this disorder are not well established, particularly regarding the influence of age at onset, disease duration, and phenotypic complexity on treatment response.

Here, we describe an adult‐onset case of *PRKRA*‐related dystonia with sustained benefit from globus pallidus internus DBS (GPi‐DBS) and contextualize our findings alongside previously reported cases to explore the relationship between clinical phenotype, surgical and quality‐of‐life outcomes.

A 47‐year‐old man from Brazil presented with a 12‐year history of abnormal upper‐limb posturing. He had no relevant past medical history. At age 35, he developed dystonic postures that progressively spread to the cervical and paraspinal muscles, leading to severe gait impairment and frequent falls. Notably, the patient exhibited a positive geste antagoniste, as placing his hand on the occipital region temporarily alleviated his cervical and truncal dystonia to improve walking (Video 1). Neurological examination revealed marked hyperextension of the cervicothoracic spine and generalized dystonia most prominent in the cervical region, speech, face, and upper limbs (Fig. S1). Subtle rigidity was noted in the upper limbs, without bradykinesia or tremor. Cognitive assessment was unremarkable.

**Video 1:** Visual comparison of clinical status before and after deep brain stimulation (DBS) implantation at 2‐year follow‐up. The video also illustrates a prominent geste antagoniste (sensory trick); tactile stimulation of the occipital area provides temporary mitigation of cervical and truncal dystonia, leading to an immediate, short‐term improvement in the patient's gait. Postoperatively, the video highlights the patient's ability to ambulate without difficulty following DBS implantation.

He is the first‐born child of consanguineous parents and has unaffected siblings. There was no family history of similar symptoms. No exposure to medications associated with secondary dystonia was identified. Brain magnetic resonance imaging (MRI) was unremarkable, and an extensive workup for Wilson's disease and other acquired causes was negative. An l‐dopa trial (400 mg/day) was ineffective, and botulinum toxin injections provided only minimal benefit. Given the progressive course, genetic testing was performed. An Next‐Generation Sequencing (NGS) panel identified a homozygous pathogenic *PRKRA* variant (NM_003690.5: c.665C>T; p.Pro222Leu), confirming the diagnosis of DYT16.

Due to severe disability and resistance to medical therapy, the patient was referred for GPi‐DBS. Bilateral electrodes were implanted without perioperative complications. Initial programming was performed with conservative monopolar settings. Details on the stimulation's parameters are described in Supporting Information S1. Over a 2‐year follow‐up, the patient experienced marked clinical improvement, including the resolution of truncal dystonia and significant gait improvement (Video 1). Quality of life improved (Brief World Health Organization Quality of Life Questionnaire (WHOQOL‐BREF): 74 preoperatively vs. 90 postoperatively), Burke–Fahn–Marsden Dystonia Rating Scale (BFMDRS) motor scores showed substantial reduction, ranging from 42 to 13 [69%], and medication burden was also reduced (Supporting Information S1).

Among cases with available genetic data,^7^, ^8^ 3 patients harbored the same homozygous c.665C>T (p.Pro222Leu) variant. DBS yielded favorable outcomes in patients who initially presented with focal dystonia that later progressed to involve speech and ultimately generalized dystonia (Table S1). When analyzing our case with previously reported patients, a sustained improvement is observed. Both juvenile‐ and adult‐onset cases demonstrate meaningful motor improvement following GPi‐DBS, evidenced by a statistically significant reduction in BFMDRS motor subscores, with an average improvement of 54% from baseline (*P* < 0.001, Fig. 1A). Statistical correlation suggests that although higher baseline motor impairment is associated with greater absolute improvement (*R* ^2^ = 0.64, Fig. 1B), disease duration was not a significant determinant of outcome (*R* ^2^ = 0.07, Fig. 1C). This indicates that long‐standing disease does not preclude a favorable surgical response, suggesting that GPi‐DBS remains a viable intervention even in the later stages of the condition.^9^, ^10^

**FIG. 1:**
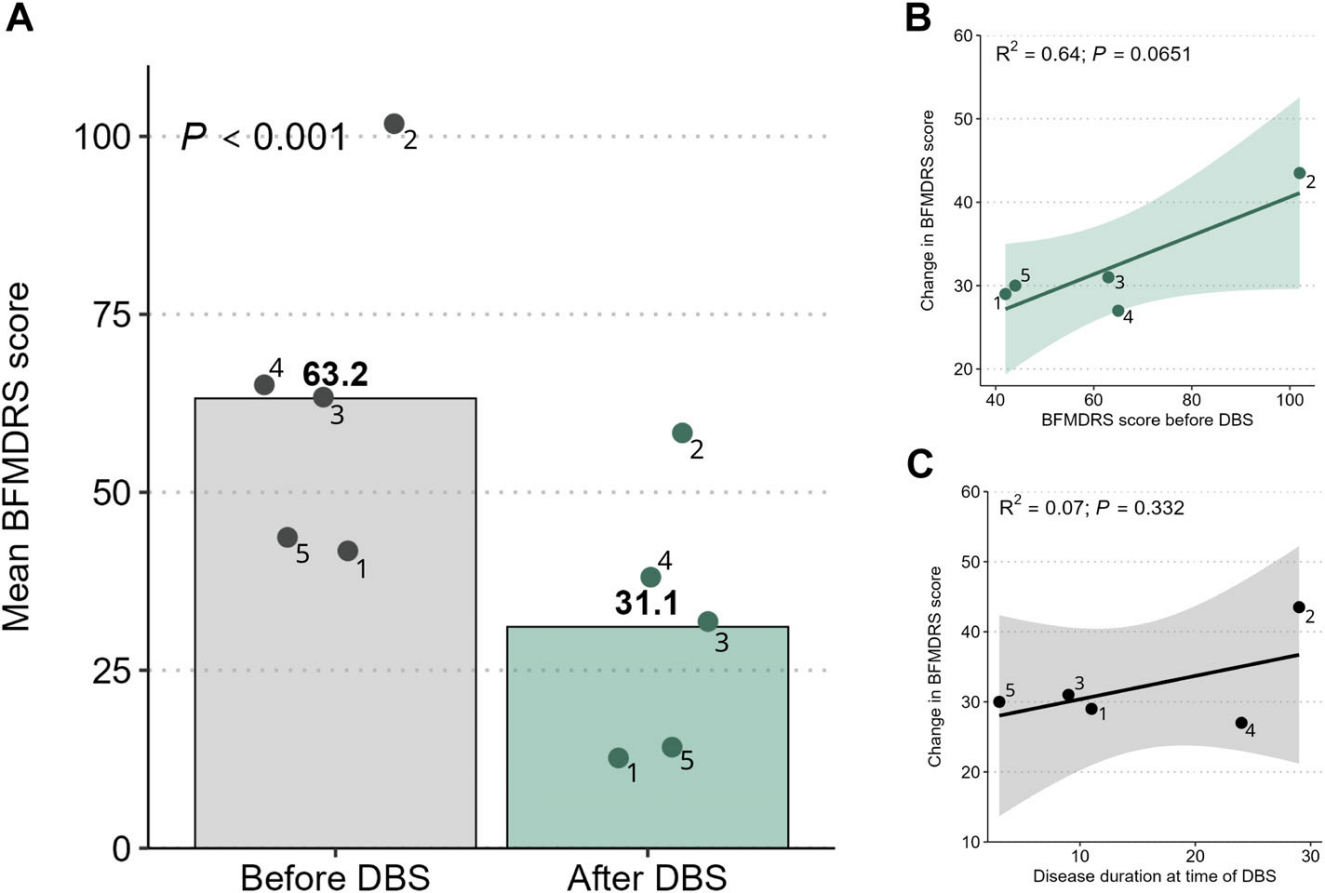
Globus pallidus internus deep brain stimulation (GPi‐DBS) efficacy outcomes and clinical correlations of all cases (n = 5) described in the literature, including the current case. (**A**) Group mean Burke–Fahn–Marsden Dystonia Rating Scale (BFMDRS) score before versus after GPi‐DBS surgery. Paired *t* test showed a significant change in motor symptoms after surgery (mean difference = −32.1 points, *P*‐value = 0.0003921), with an average 54% decrease from baseline value. Distributions were tested for normality with the Shapiro–Wilk test. (**B**) Greater baseline motor impairment was strongly associated with a greater absolute reduction in BFMDRS score after surgery (*R* ^2^ = 0.64, *P*‐value marginally significant). (**C**) Linear regression analysis showed no relation between change in BFMDRS score and disease duration at the time of surgery (*R* ^2^ = 0.07, *P*‐value = 0.332).

Quality of life was assessed using the WHOQOL‐BREF before and after DBS. Over 2 years following DBS, improvements were observed across all four WHOQOL‐BREF domains. The physical domain increased from 6.86 to 10.86 (+58.3%), the psychological domain from 11.33 to 14.67 (+29.4%), the social relationships domain from 10.67 to 13.33 (+25.0%), and the environmental domain from 12.50 to 15.50 (+24.0%). These findings indicate a broad improvement in patient‐reported quality of life following DBS, with the largest relative change observed in the physical domain.

Taken together, these observations suggest that *PRKRA*‐related dystonia should be recognized among the genetic dystonias responsive to GPi‐DBS, particularly in patients harboring the p.Pro222Leu variant. In addition, quality of life is highlighted as a good outcome for DYT‐*PRKRA* for the first time in literature.

## Disclosures

**Ethical Compliance Statement:** This study was submitted to the Institutional Ethics Committee (IRB) of Hospital de Clinicas de Porto Alegre in accordance with national and international ethical standards. Written informed consent was obtained from the patient for publication of this case report and any accompanying images. We confirm that we have read the journal's position on issues involved in ethical publication and affirm that this work is consistent with those guidelines.

**Funding Sources and Conflict of Interest:** No specific funding was received for this work. The authors declare that there are no conflicts of interest relevant to this work. Author disclosures are available in the Supporting Information.

## Supporting information


**Data S1.** COI_disclosure.

**Figure S1.** Topographical distribution and severity of dystonia based on the Burke–Fahn–Marsden Dystonia Rating Scale (BFMDRS) motor score. Color coding reflects regional severity. (**A**) Pre‐deep brain stimulation (DBS): the darkest shading indicates the most severely affected areas, with maximal involvement of the trunk (16/16), speech/swallowing (16/16), cervical/craniofacial and (6/8), and milder involvement of the arms (4/16). (**B**) Post‐DBS: trunk (4/16), speech/swallowing (4/16), cervical/craniofacialand (1/8), and arms (4/16). Created with Nano Banana and Adobe Illustrator.

**Table S1**. Summary of clinical characteristics of DYT16 (DYT‐PRRKA) submitted to globus pallidus internus deep brain stimulation (GPi‐DBS) in the literature. AO, age of onset; AS, age at DBS surgery; BFM, Burke–Fahn–Marsden Dystonia Scale; D, disability; S, severity.

**Supporting information S1.** Details regarding deep brain stimulation (DBS) and reduction in medication burden were described to better understand our patient's clinical outcome.

## Data Availability

The data that support the findings of this study are available on request from the corresponding author. The data are not publicly available due to privacy or ethical restrictions.

## References

[1] Albanese A , Bhatia KP , Fung VSC , et al. Definition and classification of dystonia. Mov Disord 2025;40:1248–1259. 10.1002/mds.30220 PMC12273609 40326714

[2] Albanese A , Romito LM , Calandrella D . Therapeutic advances in dystonia. Mov Disord 2015;30:1547–1556. 10.1002/mds.26384 26301801

[3] Indelicato E , Carmona‐Hidalgo B , Quintero J , et al. Efficacy of deep brain stimulation for the treatment of monogenic dystonia symptoms: a systematic review. Eur J Neurol 2026;33:e70490. 10.1111/ene.70490 PMC12809714 41543040

[4] Camargos S , Scholz S , Simón‐Sánchez J , et al. DYT16, a novel young‐onset dystonia‐parkinsonism disorder: identification of a segregating mutation in the stress‐response protein PRKRA. Lancet Neurol 2008;7(3):207–215. 10.1016/S1474-4422(08)70022-X 18243799

[5] Zech M , Castrop F , Schormair B , et al. DYT16 revisited: exome sequencing identifies PRKRA mutations in a European dystonia family. Mov Disord 2014;29(12):1504–1510. 10.1002/mds.25981 25142429

[6] Casagrande SCB , Listik C , Coelho DB , et al. Deep brain stimulation in patients with isolated generalized dystonia caused by PRKRA mutation. Mov Disord Clin Pract 2019;6(7):616–618. 10.1002/mdc3.12811 PMC6749865 31538099

[7] Quadri M , Olgiati S , Sensi M , et al. PRKRA mutation causing early‐onset generalized dystonia‐parkinsonism (DYT16) in an Italian family. Mov Disord 2016;31(5):765–767. 10.1002/mds.26583 26990861

[8] Ribeiro JA , Sousa M , Alonso I , Moreira F , Pereira R , Palavra F . PRKRAP1 pseudogene complicating the diagnosis of young‐onset dystonia due to PRKRA gene disease‐causing variants (DYT‐PRKRA). Mov Disord Clin Pract 2022;9(5):703–706. 10.1002/mdc3.13426 PMC9274369 35844287

[9] Lange LM , Junker J , Loens S , et al. Genotype‐phenotype relations for isolated dystonia genes: MDSGene systematic review. Mov Disord 2021;36(5):1086–1103. 10.1002/mds.28485 33502045

[10] Brüggemann N , Kühn A , Schneider SA , et al. Short‐ and long‐term outcome of chronic pallidal neurostimulation in monogenic isolated dystonia. Neurology 2015;84(9):895–903. 10.1212/WNL.0000000000001312 PMC6170184 25653290

