# Supplementary material for: Adult‐Onset Dystonia Related to PRKRA (DYT16): A Predictor of Favorable Deep Brain Stimulation Outcome
Source: Mov Disord Clin Pract. 2026 Sep 27:10.1002/mdc3.70837. Online ahead of print. doi: 10.1002/mdc3.70837 (PMC13617316; doi:10.1002/mdc3.70837)
Supplement: Supplementary file 1 [file MDC3-9999-0-s001.pdf]

Table S1. Summary of clinical characteristics of DYT-16 (DYT-PRRKA) submitted to GPi-DBS in the literature

N	Sex	AO (y)	Age DBS Surgery (AS) (y)	Origin	Initial symptoms	Current symptoms	Parkinsonism	Genotype	Treatment	DBS target	Other surgeries	Time between AO and AS (y)	Improvement of BFM scale (S/D)	References
1	M	38	49	Brazil	Focal dystonia (upper limb)	Generalized dystonia	No	Homozygous c. 665C>T (Pro222Leu)	Levodopa/benser azide; biperiden; baclofen; botulin	Bilateral GPi DBS		11	69%/NR	Current case
2	M	6	35	Brazil	Hand and vocal dystonia	Generalized dystonia	No	Not reported	Levodopa; anticholinergic; botox	Bilateral GPi DBS	Unilateral palidotomy at the age of 31	29	42% / 50%	Casagrande *et al*
3	F	7	16	Brazil	Focal dystonia (right lower lim)	Generalized dystonia	No	Not reported	Conventional oral medications; botulin toxin	Bilateral GPi DBS		9	49% / 57%	Casagrande *et al*
4	M	4	28	Italy	Spasmodic dysphonia, dysarthria	Generalized dystonia	Yes	Homozygous c. 665C>T (Pro222Leu)	Levodopa; anticholinergic; botox;	Bilateral GPi DBS		24	41.5% / 59%	Quadri *et al*
5	M	8	11	Portugal	Focal dystonia (right lower limb)	Generalized dystonia	No	Homozygous c. 665C>T (Pro222Leu)	Levodopa/carbido pa; clonazepam; baclofen;	Bilateral GPi DBS		3	68%/NR	Ribeiro *et al*

Table S1: AO: age of onset; AS: age at DBS surgery; BFM: Burke-Fahn-Marsden Dystonia Scale; D: disability; S: severity
