# Supplementary material for: Adult‐Onset Dystonia Related to PRKRA (DYT16): A Predictor of Favorable Deep Brain Stimulation Outcome
Source: Mov Disord Clin Pract. 2026 Sep 27:10.1002/mdc3.70837. Online ahead of print. doi: 10.1002/mdc3.70837 (PMC13617316; doi:10.1002/mdc3.70837)
Supplement: Supplementary file 3 [file MDC3-9999-0-s004.pdf]

DBS STIMULATION

Stimulation began bilaterally with the following parameters: most ventral contact with amplitude: 1.0 mA, pulse width (pw): 60 μs, and frequency 130 Hz. Subsequent improvements of amplitude were achieved, however due to stimulation-induced dysarthria, pulse width was downgraded to 30μs. Later, advanced stimulation was necessary to maintain adequate effect: Left GPi with stimulation on ventral contact with 2mA, pw at 60μs frequency of 104Hz and dorsal contact with 2mA, pw at 80μs and frequency of 130Hz. Both contacts with directionality towards the lateral side. Right GPi with ring stimulation on ventral contact with 3,1mA of amplitude, pw at 60μs and frequency of 130Hz.

MEDICATION BURDEN

Prior to GPi-DBS implantation (in 2022), the patient presented with severe dystonic symptoms that required aggressive multi-drug therapy and high-dose interventions. His baseline oral medication regimen consisted of levodopa 400 mg/day, biperiden 4 mg/day, clonazepam 2 mg/day, and baclofen 30 mg/day. Additionally, he was undergoing regular adjuvant (each 4-5 months) therapy with high-dose botulinum toxin injections, receiving a total of 500 UI distributed across the paravertebral muscles (150 UI bilaterally), splenius capitis (50 UI bilaterally), and sternocleidomastoid (50 UI bilaterally). Following successful GPi-DBS surgery, the patient’s medication burden was substantially reduced due to marked clinical improvement. Levodopa and biperiden were completely discontinued, and botulinum toxin sessions were no longer required. At the most recent follow-up, his remaining oral maintenance therapy was minimized to clonazepam 1 mg at night and paroxetine 25 mg/day. Baclofen was significantly decreased to 10 mg twice daily and is currently undergoing progressive tapering, reflecting sustained surgical benefit while safely managing mild residual symptoms.
